# The Impact of Low Muscle Mass Definition on the Prevalence of Sarcopenia in Older Australians

**DOI:** 10.1155/2014/361790

**Published:** 2014-07-03

**Authors:** Solomon Yu, Sarah Appleton, Robert Adams, Ian Chapman, Gary Wittert, Thavarajah Visvanathan, Renuka Visvanathan

**Affiliations:** ^1^Aged and Extended Care Services, The Queen Elizabeth Hospital, Central Adelaide Local Health Network and Adelaide Geriatric Training and Research with Aged Care (G-TRAC) Center, School of Medicine, University of Adelaide, Level 8B Main Building, 21 Woodville Road, Woodville South, Adelaide, SA 5011, Australia; ^2^The Health Observatory, Discipline of Medicine, School of Medicine, Faculty of Health Science, University of Adelaide, Adelaide, SA 5000, Australia; ^3^Discipline of Medicine, School of Medicine, Faculty of Health Science, University of Adelaide, Adelaide, SA 5000, Australia; ^4^Department of Anaesthesia, The Queen Elizabeth Hospital, Central Adelaide Local Health Network, Adelaide, SA 5000, Australia

## Abstract

*Background.* Sarcopenia is the presence of low muscle mass and low muscle function. The aim of this study was to establish cutoffs for low muscle mass using three published methods and to compare the prevalence of sarcopenia in older Australians. *Methods.* Gender specific cutoffs levels were identified for low muscle mass using three different methods. Low grip strength was determined using established cutoffs of <30 kg for men and <20 kg for women to estimate the prevalence of sarcopenia. *Results.* Gender specific cutoffs levels for low muscle mass identified were (a) <6.89 kg/m^2^ for men and <4.32 kg/m^2^ for women, <2 standard deviation (SD) of a young reference population; (b) <7.36 kg/m^2^ for men and <5.81 kg/m^2^ for women from the lowest 20% percentile of the older group; and (c) <−2.15 for men and <−1.42 for women from the lowest 20% of the residuals of linear regressions of appendicular skeletal mass, adjusted for fat mass and height. Prevalence of sarcopenia in older (65 years and older) people by these three methods for men was 2.5%, 6.2%, and 6.4% and for women 0.3%, 9.3%, and 8.5%, respectively. *Conclusions.* Sarcopenia is common but consensus on the best method to confirm low muscle mass is required.

## 1. Introduction

Sarcopenia commonly affects older people and is characterized by loss of both muscle mass and strength [1, 2]. Sarcopenia is associated with disability, a loss of independence, and reduced quality of life [3]. In one American study, sarcopenia and its consequences were estimated to cost the US healthcare system US$18 billion [4]. Sarcopenia is therefore a costly issue to the healthcare system [4, 5].

The European Working Group on Sarcopenia in Older People (EWGSOP) has recently defined sarcopenia as a combination of both low muscle mass and low muscle function [1]. Grip strength is one method to assess muscle function [1]. Low grip strength cutoffs of <30 kg for men and <20 kg for women are recommended and derived from receiver operating characteristic (ROC) curves predicting walking speeds slower than 0.8 m/s [6]. Appendicular skeletal muscle mass (ASM) is commonly assessed using dual absorptiometry X-ray assessment (DXA). The EWGSOP identifies three different methods to define low muscle mass [1]. With the oldest method, gender specific cut-off values for low muscle mass are derived from a younger reference group (<2 standard deviation, age 18–40 years) and cut-off values of <7.26 kg/m^2^ for men and <5.50 kg/m^2^ for women were reported in the original paper [2]. With the second method, cut-off points for low muscle mass are derived from gender specific lowest 20% of a predictive population, thus circumventing the need for a younger reference group [7]. Cut-off points similar to those identified by the Newman and colleagues have been reported, <7.23 kg/m^2^ for men and <5.67 kg/m^2^ for women [7, 8]. The third method adjusts for fat mass and is derived from the gender specific lowest 20% of the distribution of residuals of the linear regression on appendicular lean mass adjusted for fat mass and height and cutoffs of <−2.29 kg for men and <−1.73 kg for women are reported [7].

To date, there have only been three studies in Australia investigating the prevalence of low muscle mass but only one has reported on the prevalence of sarcopenia (i.e., low muscle mass and low muscle strength) in the community [9–11]. Scott et al. reported a 5% prevalence of sarcopenia in those aged 50–79 years and using the lowest 20% distribution of the predictive population to identify the cut-off points for both low muscle mass and low grip strength [9]. In a second Australian study, cut-off points of <4.85 kg/m^2^ derived from a young reference group were used to identify that 3.2% of older women residing in low level aged care have sarcopenia [10]. The third Australian study examined the prevalence of low ASM in older (≥70 years) men living in the community using the linear regression and the gender specific lowest 20% method and reported a prevalence rate ranging from 15% in those aged 70 to 74 years to 26% for those aged 80–84 years and increasing to 45% for those aged 85–89 years [11].

To date, no study in Australia has examined the prevalence of sarcopenia in both men and women and compared all three methods to identify low muscle mass. The aims of this study were to firstly establish gender specific cut-off points for low skeletal muscle mass using the three methods as identified by the EWGSOP and then report the prevalence of sarcopenia in older (aged 65 years and older) Australians living in the community.

## 2. Methods

### 2.1. Study Cohorts

**Figure 1:**
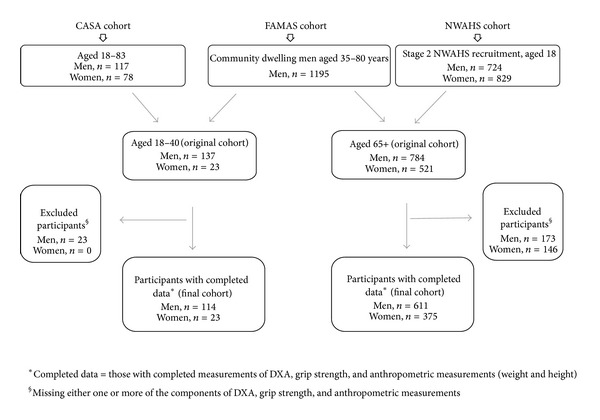
Cohorts combined to develop the younger reference (aged 18–<40) and older study group (aged 65+).

Three cohorts were investigated in this study: The Cytokine, Adiposity, Sarcopenia and Ageing Study (CASA), the North West Adelaide Health Study (NWAHS), and the Florey Adelaide Male Ageing Study (FAMAS) [12–14]. The three cohorts were combined to derive two broad population groups: younger reference population (aged 18–40 years; CASA and FAMAS) and older group (aged ≥65; FAMAS and NWAHS) (see Figure 1). For the purpose of this study, only those participants with a complete set of information on weight, height, grip strength, and DXA were included in the analysis.

The methodology of recruitment was similar for all three cohorts and has been described in detail elsewhere [12–14]. Ethical approval was obtained from the Central Northern Adelaide Health Service Ethics of Human Research Committee. All participants in the three cohort studies provided written, informed consent. Briefly, all households in the northern and western region of Adelaide with a telephone number listed in the Electronic White Pages were eligible for selection into the study. Selected households were sent an approach letter and brochure informing them about the study. The person who was last to have a birthday and aged 18 years or older was invited to participate in a short telephone interview. Interviews were conducted using computer-assisted telephone interview (CATI) technology. Selected persons were deemed “nonreplaceable” and, if the selected person was not available, interviews were not conducted with alternative household members. Up to six telephone calls were made to each household before the selected individual was classified as noncontactable. Respondents to the telephone interview were asked a number of health-related and demographic questions. Following the recruitment interview, respondents were invited to make an appointment to attend clinic for biomedical examination and investigations.

*NWAHS*. 4060 adults were included in the baseline biomedical examination between December 1999 and July 2003. 3566 participants attended the followup (median 4 years) between May 2004 and February 2006. Of these, a total of 1553 participants aged 65 years and older (men = 724, women = 829) were included in the analysis [12].

*FAMAS*. 1195 community dwelling men aged between 35 and 80 years from the north west regions of Adelaide were recruited between August 2002 and April 2005. Of these, 295 men were aged 65 years and older [13].

*CASA*. Healthy subjects aged 18 to 83 years (*n* = 195) were recruited from the western suburbs of Adelaide (2005–mid-2007). In this study, as the aim was to recruit a “healthier” population and so there were additional criteria. To participate in this study, subjects had to be 18 years and older, be able to comply with the study protocol, and be weight stable over the preceding three months. Those with a serious medical illness, inflammatory disease, an acute illness in the previous three months or in the two weeks following blood sampling, unable to stop medications for three days prior to blood sampling, in receipt of vaccinations, and pregnant were excluded from the study [14].

### 2.2. Measurements

*Anthropometry*. Height (m) was measured with shoes off to the nearest 0.1 cm. Weight (kg) was measured wearing light clothing to the nearest 0.1 kg. Body mass index (BMI, weight/height^2^) was calculated. Three measurements of the waist and hip were taken and the mean for each was calculated [12].

*Grip Strength*. Grip strength (kg) was measured three times with each dominant hand using a grip dynamometer (Lafayette Instrument Company, IN, USA [CASA and NWAHS], Smedley, Chicago, IL [FAMAS]) while subjects were sitting with their arm supported by a horizontal surface. The mean of the three readings was used in this study [15].

*Dual Energy X-Ray Absorptiometry (DXA)*. Appendicular skeletal muscle mass (ASM) in this study was defined as the sum of lean soft-tissue masses for arms and legs, assuming that all nonfat and nonbone tissue are skeletal muscle.* CASA*: ASM was determined using a Lunar PRODIGY whole-body scanner (GE Medical Systems, Madison, WI) in conjunction with Encore 2002 software.* NWAHS* and* FAMAS*: A Lunar PRODIGY scanner (GE Medical Systems, Madison, WI) in conjunction with Encore 2002 software and a DPX+ (GE Medical Systems, Madison, WI) scanner in conjunction with LUNAR software version 4.7e were used. Cross-calibration analysis reported no significant differences between the 2 machines [16].

### 2.3. Statistical Analysis

SPSS 19 for Windows software (SPSS, Inc., Chicago, IL) was used for statistical analysis. Descriptive data was expressed as mean ± standard deviation (SD). Independent two-sample* t*-test was used to assess the mean difference in the characteristics variables between men and women. Low muscle mass was identified using the three different methods: (a) Baumgartner's method whereby cut-off values of ASM were <2 standard deviation (SD) of a young reference population, (b) the 20% gender specific method where cutoffs were derived for the lowest 20% of the older study population, and (c) the linear regression method where the lowest 20% of residual of the linear regression models of ASM adjusting for fat mass and height in men and women were applied to the older study population to derive cut points. As walk speed was not available within the NWAHS cohort, grip strength was used to determine muscle function and cutoffs of <30 kg for men and <20 kg for women were applied [6]. *P* < 0.05 was considered statistically significant.

## 3. Results

Figure 1 illustrates the flow diagram in establishing the two study populations from the three cohorts. For the young reference group, from the CASA and FAMAS cohort, there were a total of 137 men and 23 women aged 18–40 years. Of these, 23 men were excluded because of insufficient data. There were no statistically significant differences between the original and final cohorts in terms of age (35.7 ± 4.9 versus 35.5 ± 5.3 years, *P* = 0.75), weight (88.0 ± 16.3 versus 87.7 ± 15.9 kg, *P* = 0.99), height (1.8 ± 0.1 versus 1.8 ± 0.1 m, *P* = 0.98), BMI (27.9 ± 4.6 versus 27.8 ± 4.6 kg/m^2^, *P* = 0.98), % fat (26.7 ± 8.5 versus 26.7 ± 8.5%, *P* = 0.97), ASM (28.6 ± 4.3 versus 28.6 ± 4.3 kg, *P* = 0.83), SMI (9.1 ± 1.1 versus 9.1 ± 1.1 kg/m^2^, *P* = 0.85), and grip strength (52.2 ± 10.8 versus 51.6 ± 11.1, *P* = 0.68). For the older group, from the FAMAS and NWAHS cohorts, there were 784 men and 521 women (Figure 1). 173 men and 146 women were excluded because of incomplete data. Consequently, the final cohort consisted of 611 men and 375 women. Women in the original cohort were significantly older than the women in the final cohort (74.0 ± 6.3 versus 73.2 ± 6.0 years, *P* = 0.05). No age difference was noted for men (73.0 ± 6.0 versus 72.7 ± 5.7 years, *P* = 0.30). There were no statistically significant differences between the original and final cohort in terms of weight (81.8 ± 13.6 versus 81.8 ± 13.3 kg, *P* = 0.96), height (1.7 ± 0.1 versus 1.7 ± 0.1 years, *P* = 0.85), BMI (27.9 ± 4.3 versus 27.9 ± 4.2 kg/m^2^, *P* = 0.88), % fat (28.6 ± 6.9 versus 28.6 ± 6.9%, *P* = 0.95), ASM (23.9 ± 3.3 versus 24.0 ± 3.2 kg, *P* = 0.92), SMI (8.2 ± 0.9 versus 8.2 ± 0.9 kg/m^2^, *P* = 0.94), and grip strength (37.2 ± 8.9 versus 37.6 ± 8.9 kg, *P* = 0.37).

Table 1 shows the characteristics of participants in the final cohort aged 18–40 and aged 65 years and older. Comparing men to women in the younger reference group, men were significantly older (35.5 ± 5.3 versus 31.2 ± 7.3 years, *P* = 0.01), heavier (87.7 ± 15.9 versus 69.3 ± 15.3 kg, *P* < 0.001), and taller (1.8 ± 0.1 versus 1.7 ± 0.1 m, *P* < 0.001) and had higher BMI (27.8 ± 4.6 versus 25.5 ± 5.5 kg/m^2^, *P* = 0.03) and SMI (9.1 ± 1.1 versus 6.7 ± 1.2 kg/m^2^, *P* < 0.001) than women. Similar to the younger population group, older men were significantly heavier (81.8 ± 13.3 versus 69.4 ± 12.4 kg, *P* < 0.001), and taller (1.7 ± 0.1 versus 1.6 ± 0.1 m, *P* < 0.001) and with higher values for ASM (24.0 ± 3.2 versus 16.1 ± 2.4 kg, *P* < 0.001) and SMI (8.2 ± 0.9 versus 6.4 ± 0.8 kg/m^2^, *P* < 0.001) than women. Interestingly, there was no difference in the BMI (27.9 ± 4.2 versus 27.8 ± 4.7 kg/m^2^, *P* = 0.79) between the older men and women. The spread of various chronic conditions was shown in Table 1 with higher prevalence of chronic conditions amongst the older population compared to the younger population.

In men, low grip strength (Table 2) was noted in approximately 14% of men aged between 65 and less than 80 years and almost half of men aged 80 years and older. A higher proportion of women (i.e., 33.5%) between 65 years and less than 80 years had low grip strength compared to men. Similarly, 63% of women aged 80 years and older had low grip strength and this was higher in proportion within the same age group of men.

The cut-off points (Table 2) for low muscle mass identified were as follows:

- <6.89 kg/m^2^ for men and <4.32 kg/m^2^ for women by Baumgartner's method;
- <7.36 kg/m^2^ for men and <5.81 kg/m^2^ for women by the 20% gender specific method;
- <−2.15 for men and <−1.42 for women using the linear regression method. The linear regression model was ASM (kg) = −18.24 + 23.09 × height (m) + 0.11 × total fat mass for men and ASM (kg) = −15.84 + 18.18 × height (m) + 0.11 × total fat mass for women.

The prevalence of low muscle mass ranged between 7 and 18% for men aged between 65 and 80 years but increased to between 12 and 29.6% for men aged 80 years and older (Table 2). However, for women, there was no increase in the reported prevalence with increasing age with the prevalence of low muscle mass ranging from 0 to 20.1% in those aged between 65 and <80 years and remaining between 1.6–19.4% in those aged 80 years and older. The prevalence reported by the 20% gender specific method and linear regression method was similar and much higher than the prevalence reported by Baumgartner's method.

**Figure 2:**
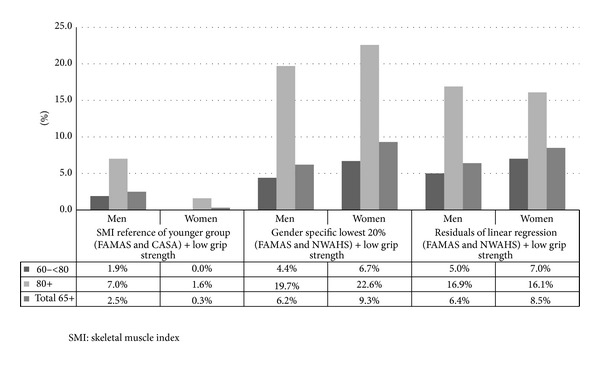
Comparison of prevalence rate of sarcopenia as defined by EWGSOP, by using different methods of SMI cut points derivation with a low grip strength (<30 kg for men and <20 kg for women).

Figure 2 shows that the prevalence of sarcopenia was higher in men (7–19.7%) and women (1.6–22.6%) aged 80 years and older compared to men (1.9–5.0%) and women (2.5–7.0%) aged between 65 and <80 years. The prevalence of sarcopenia in people aged 65 years and older in this study was between 2.5% and 6.4% for men and between 0.3% and 9.3% for women. The overall prevalence of sarcopenia as estimated by Baumgartner's method, the lowest 20% method, and linear regression method was 1.6%, 7.4%, and 7.2%, respectively.

## 4. Discussion

The key finding from this study is that in combination with grip strength, different methods of determining low muscle mass result in different sarcopenia prevalence. The cut-off points for low muscle mass derived by the gender specific lowest 20% method and the linear regression method yielded similar prevalence rates for low muscle mass and sarcopenia. Also, the cutoffs generated by these two methods, in this study, were similar to those reported by EWGSOP [1]. However, the cutoffs derived by the Baumgartner method (<6.89 kg/m^2^ for men and <4.32 kg/m^2^ for women), in this study, were much lower than that previously reported (<7.26 kg/m^2^ for men and <5.50 kg/m^2^ for women) [2]. Our findings of a lower cutoff than that previously reported was similarly noted in an Australian study of women (<4.85 kg/m^2^) [10]. Researchers from Korea have recently reported similar SMI cut-off values (6.58 kg/m^2^ for men and 4.59 kg/m^2^ for women) [17]. The mean ASM for the younger reference population in this study was lower than that reported in the Baumgartner (28.6 kg versus 30.6 kg for men and 18.4 kg versus 20.9 kg) study and this is potentially contributing to the difference in the reported cut-off values [2]. Importantly, the sample size making up the younger reference population in our study was small and so there is a need to derive cutoffs from a larger cohort of younger people before firm conclusions can be reached.

Using the lowest 20% method and linear regression method to define low muscle mass, the prevalence of sarcopenia reported in this study was approximately 6.2% for men and 9% of women aged 65 years. To the best of our knowledge, there has only been one other Australian study which used the lowest 20% method to define low muscle mass [9]. In that study, the overall sarcopenia prevalence rate was 5% [9]. We observed a higher overall prevalence rate at 7.6% and this is likely due to older age group in our study population compared with the population in the other Australian study (72.7 ± 5.7 versus 61.7 ± 7.1 years in men and 73.2 ± 6.0 versus 61.0 ± 6.8 years in women) [9].

Consistent with other studies, the prevalence of low muscle mass increased with age in men and was higher in those aged 80 years and older compared to those between 65 and <80 years using all three methods [18]. However, in women, this relationship was not seen with the linear regression method, which also accounts for fat mass. Fat mass reduces with increasing age in women but not in men [19]. In this study, the prevalence of low grip strength increased with age in both men and women. A greater proportion of women however met the criteria of low grip strength compared to men in older age. It is well known that a decline in sex hormones with increasing age (andropause and menopause) contributes to decline in strength [20].

Both the FAMAS and the NWAHS cohorts did not include subjects from residential care facilities where the prevalence of sarcopenia is likely higher. The requirement for subjects to attend a hospital based clinic also made it very likely that frail individuals may have been less likely to participate. Therefore, the reported prevalence in this study is likely to be an underestimate of the true prevalence of sarcopenia in the community. Subjects enrolled in these studies were predominantly Caucasian and so the findings from this study are not generalizable to the wider multicultural Australian population. Ethnic specific cutoffs need to be determined and future research including different ethnic population groups is important.

## 5. Conclusion

To conclude, the prevalence of sarcopenia varies depending on the method used to estimate the cut-off values for low muscle mass. Therefore, a consensus is required to identify the preferred method to define Sarcopenia. This will allow for pooling of research data. However, sarcopenia is common in the community. Given that sarcopenia is linked to morbidity and costs [4], early recognition and intervention through exercise and nutritional programs may contribute to healthy ageing outcomes and so a reduction in health costs [21].

## Figures and Tables

**Table 1:** Characteristics of subjects from the younger reference group and older adults (aged ≥ 65) in the NWAHS and FAMAS included in the analysis.

Characteristics	Younger reference population			Older study population		
	18 +< 40 years (FAMAS and CASA)			65+ years (NWAHS and FAMAS)		
	Men (*n* = 117) mean (SD)	Women (*n* = 23) mean (SD)	*P* values	Men (*n* = 611) mean (SD)	Women (*n* = 375) mean (SD)	*P* values
Age (SD), years	35.5 (5.3)	31.2 (7.3)	0.01	72.7 (5.7)	73.2 (6.0)	0.21
Weight (SD), kg	87.7 (15.9)	69.3 (15.3)	<0.001	81.8 (13.3)	69.4 (12.4)	<0.001
Height (SD), m	1.8 (0.1)	1.7 (0.1)	<0.001	1.7 (0.1)	1.6 (0.1)	<0.001
BMI (SD), kg/m^2^	27.8 (4.6)	25.5 (5.5)	0.03	27.9 (4.2)	27.8 (4.7)	0.79
% Fat	26.7 (8.5)	29.9 (11.6)	0.22	28.6 (6.9)	40.2 (6.9)	<0.001
ASM (SD), kg	28.6 (4.3)	18.4 (4.1)	<0.001	24.0 (3.2)	16.1 (2.4)	<0.001
SMI (SD), kg/m^2^	9.1 (1.1)	6.7 (1.2)	<0.001	8.2 (0.9)	6.4 (0.8)	<0.001
Chronic conditions	%	%		%	%	
Cardiovascular Disease	1.7	0.0	0.54	24.1	17.8	0.019
Diabetes	1.7	0.0	0.54	24.4	19.1	0.050
Hypertension	27.6	4.5	0.02	77.3	69.7	0.007
Hypercholesterolemia	44.7	13.6	0.06	31.1	50.3	<0.001
Arthritis	0.9	0.0	0.66	33.7	61.5	<0.001
Number of prescribed medications						
0	92.2	54.5	<0.001	15.1	6.3	0.02
1–3	7.8	45.5		37.1	39.7	
4–6	0.0	0.0		25.8	32.9	
≥7	0.0	0.0		22.0	21.1	

SMI, skeletal muscle index; ASM, appendicular skeletal muscle mass; BMI, body mass index; SD, standard deviation; NS, not significant (*P* > 0.05); NA, not applicable; cardiovascular disease, ischemic heart disease, acute myocardial infarction, stroke, and angina; diabetes, self-reported, Dr diagnosed, FPG ≥ 7.0 mmol/L, or HbA1c ≥ 6.5%; hypertension, BP ≥ 140/90, or already on treatment; hypercholesterolaemia, serum total cholesterol ≥5.5 mmol/L; arthritis, self-reported osteo- or rheumatoid.

**Table 2:** The prevalence of low muscle mass and low grip strength in the North West Adelaide Health Study (NWAHS) and Florey Adelaide Male Ageing Study (FAMAS) based upon dual absorptiometry X-ray assessments of appendicular skeletal muscle mass.

	Low grip strength (*n*%)	Low SMI (*n*%)	Low SMI (*n*%)	Low SMI (*n*%)
	EWGSOP Criteria [6]	<2 SD below mean of younger reference group (FAMAS and NWAHS) (Table 1)	Gender specific lowest 20% of study group (FAMAS and NWAHS)	Residuals of linear regression on appendicular lean mass adjusted for fat and height (FAMAS and NWAHS)
NWAHS + FAMAS men				
Cut-offs	<30 Kg	<6.89 Kg/m^2^	<7.36 Kg/m^2^	<−2.15 Kg
65 −< 80 (*n* = 540)	78 (14.4)	38 (7.0)	92 (17.0)	101 (18.7)
80+ (*n* = 71)	32 (45.1)	9 (12.7)	29 (40.8)	21 (29.6)

Total 65+ (*n* = 611)	110 (18.0)	44 (7.2)	121 (19.8)	122 (20)

NWAHS female				
Cutoffs	<20.0 Kg	<4.32 Kg/m^2^	<5.81 Kg/m^2^	<−1.42 Kg
65 −<80 (*n* = 313)	105 (33.5)	0 (0)	56 (17.9)	63 (20.1)
80+ (*n* = 62)	39 (62.9)	1 (1.6)	18 (29)	12 (19.4)

Total 65+ (*n* = 375)	144 (38.4)	1 (1.6)	74 (19.7)	75 (20)
